# Automated Real-Time Collection of Pathogen-Specific Diagnostic Data: Syndromic Infectious Disease Epidemiology

**DOI:** 10.2196/publichealth.9876

**Published:** 2018-07-06

**Authors:** Lindsay Meyers, Christine C Ginocchio, Aimie N Faucett, Frederick S Nolte, Per H Gesteland, Amy Leber, Diane Janowiak, Virginia Donovan, Jennifer Dien Bard, Silvia Spitzer, Kathleen A Stellrecht, Hossein Salimnia, Rangaraj Selvarangan, Stefan Juretschko, Judy A Daly, Jeremy C Wallentine, Kristy Lindsey, Franklin Moore, Sharon L Reed, Maria Aguero-Rosenfeld, Paul D Fey, Gregory A Storch, Steve J Melnick, Christine C Robinson, Jennifer F Meredith, Camille V Cook, Robert K Nelson, Jay D Jones, Samuel V Scarpino, Benjamin M Althouse, Kirk M Ririe, Bradley A Malin, Mark A Poritz

**Affiliations:** ^1^ BioFire Diagnostics Salt Lake City, UT United States; ^2^ bioMérieux USA Durham, NC United States; ^3^ Hofstra Northwell School of Medicine Hempstead, NY United States; ^4^ Department of Pathology and Laboratory Medicine Medical University of South Carolina Charleston, SC United States; ^5^ Departments of Pediatrics and Biomedical Informatics University of Utah School of Medicine Salt Lake City, UT United States; ^6^ Laboratory of Microbiology and Immunoserology Department of Laboratory Medicine Nationwide Children's Hospital Columbus, OH United States; ^7^ Department of Lab Operations South Bend Medical Foundation South Bend, IN United States; ^8^ Department of Pathology New York University Winthrop Hospital Mineola, NY United States; ^9^ Clinical Microbiology and Virology Laboratory Department of Pathology and Laboratory Medicine Children's Hospital of Los Angeles Los Angeles, CA United States; ^10^ Keck School of Medicine University of Southern California Los Angeles, CA United States; ^11^ Molecular Genetics Laboratory Stony Brook University Medical Center Stony Brook, NY United States; ^12^ Department of Pathology and Laboratory Medicine Albany Medical Center Albany, NY United States; ^13^ Department of Pathology Wayne State University School of Medicine Detroit, MI United States; ^14^ Clinical Microbiology, Virology and Molecular Infectious Diseases Laboratory Department of Pathology and Laboratory Medicine Children's Mercy Hospital Kansas City, MO United States; ^15^ Department of Pathology and Laboratory Medicine Division of Infectious Disease Diagnostics Northwell Health Lake Success, NY United States; ^16^ Department of Pathology University of Utah School of Medicine Salt Lake City, UT United States; ^17^ Department of Pathology Intermountain Medical Center Murray, UT United States; ^18^ Laboratory of Microbiology University of Massachusetts Medical School-Baystate Springfield, MA United States; ^19^ Department of Pathology and Medicine Divisions of Clinical Pathology and Infectious Diseases UC San Diego San Diego, CA United States; ^20^ Department of Clinical Laboratories New York University Langone Health New York, NY United States; ^21^ Department of Pathology and Microbiology University of Nebraska Medical Center Omaha, NE United States; ^22^ Department of Pediatrics Washington University St. Louis, MO United States; ^23^ Department of Pathology and Clinical Laboratories Nicklaus Children's Hospital Miami, FL United States; ^24^ Department of Pathology and Laboratory Medicine Microbiology/Virology Laboratory Section Children's Hospital Colorado Aurora, CO United States; ^25^ Department of Laboratory Services Microbiology Section Greenville Health System Greenville, SC United States; ^26^ Northeastern University Boston, MA United States; ^27^ University of Washington Seattle, WA United States; ^28^ New Mexico State University Las Cruces, NM United States; ^29^ bioMérieux Salt Lake City, UT United States; ^30^ Department of Biomedical Informatics School of Medicine Vanderbilt University Nashville, TN United States; ^31^ BioFire Defense Salt Lake City, UT United States

**Keywords:** epidemiology, patients, privacy, communicable disease, internet, pathology, molecular

## Abstract

**Background:**

Health care and public health professionals rely on accurate, real-time monitoring of infectious diseases for outbreak preparedness and response. Early detection of outbreaks is improved by systems that are comprehensive and specific with respect to the pathogen but are rapid in reporting the data. It has proven difficult to implement these requirements on a large scale while maintaining patient privacy.

**Objective:**

The aim of this study was to demonstrate the automated export, aggregation, and analysis of infectious disease diagnostic test results from clinical laboratories across the United States in a manner that protects patient confidentiality. We hypothesized that such a system could aid in monitoring the seasonal occurrence of respiratory pathogens and may have advantages with regard to scope and ease of reporting compared with existing surveillance systems.

**Methods:**

We describe a system, BioFire Syndromic Trends, for rapid disease reporting that is syndrome-based but pathogen-specific. Deidentified patient test results from the BioFire FilmArray multiplex molecular diagnostic system are sent directly to a cloud database. Summaries of these data are displayed in near real time on the Syndromic Trends public website. We studied this dataset for the prevalence, seasonality, and coinfections of the 20 respiratory pathogens detected in over 362,000 patient samples acquired as a standard-of-care testing over the last 4 years from 20 clinical laboratories in the United States.

**Results:**

The majority of pathogens show influenza-like seasonality, rhinovirus has fall and spring peaks, and adenovirus and the bacterial pathogens show constant detection over the year. The dataset can also be considered in an ecological framework; the viruses and bacteria detected by this test are parasites of a host (the human patient). Interestingly, the rate of pathogen codetections, on average 7.94% (28,741/362,101), matches predictions based on the relative abundance of organisms present.

**Conclusions:**

Syndromic Trends preserves patient privacy by removing or obfuscating patient identifiers while still collecting much useful information about the bacterial and viral pathogens that they harbor. Test results are uploaded to the database within a few hours of completion compared with delays of up to 10 days for other diagnostic-based reporting systems. This work shows that the barriers to establishing epidemiology systems are no longer scientific and technical but rather administrative, involving questions of patient privacy and data ownership. We have demonstrated here that these barriers can be overcome. This first look at the resulting data stream suggests that Syndromic Trends will be able to provide high-resolution analysis of circulating respiratory pathogens and may aid in the detection of new outbreaks.

## Introduction

### Surveillance Landscape

The availability of real-time surveillance data that can monitor the spread of infectious diseases benefits public health [1-3]. At present, tracking of respiratory or foodborne outbreaks relies on a variety of methods ranging from automated real-time electronic reporting to manual Web entry of test results. Systems such as the Centers for Disease Control and Prevention’s (CDC) FluView [4], National Respiratory and Enteric Virus Surveillance Systems (NREVSS) [5], National Electronic Disease Surveillance System [6], Global Emerging Infections Surveillance (GEIS) [7], and others, although Web-based, still require manual entry of data from laboratories, resulting in data that are often incomplete or not current.

Syndrome-based surveillance systems [8-10] include BioSense (extraction of symptomatic data from electronic health records [11]), Google Flu (tracking of internet search queries [12] but recently discontinued [13]), and Flu Near You (voluntary reporting [14]). Additionally, numerous next generation, syndromic surveillance systems, for example, pharmacy sales records [15,16], Twitter conversations [17,18], and Wikipedia hits [19,20] have come online in the past 5 years. However, these systems cannot report the specific pathogen causing an increase in a particular set of symptoms. Finally, there are more localized efforts such as GermWatch in Utah [21] and the Electronic Clinical Laboratory Reporting System (ECLRS) in New York [22] that draw from hospital information systems (HISs) and laboratory information systems (LISs). This disparity in technologies and data collection methods results in incomplete surveillance.

### Comprehensive Testing

Comprehensive and uniform diagnostic test data will aid in the identification of potential outbreaks. A combination of broad respiratory pathogen testing and an internal electronic surveillance system enabled the rapid dissemination of data across the largest health care system in New York, the North Shore-LIJ Health System (now Northwell Health), during the influenza A H1N1-2009 pandemic in the New York City area. Pathogen-specific molecular testing permitted rapid (1) notification to state epidemiologists, (2) tracking of the virus so that health care resources could be managed effectively, and (3) evaluation of influenza diagnostics [23,24]. Today, with the threat of emerging pathogens such as Middle East respiratory syndrome coronavirus (CoV), avian influenza, enterovirus (EV) D68, and Ebola virus, real-time surveillance programs are critical [25,26].

It is not always possible to accurately diagnose the causative agents of most infectious diseases from symptoms alone because of overlapping clinical presentation. Thus, to achieve maximal utility, infectious disease surveillance systems should move beyond syndrome-based reporting and be pathogen-specific and comprehensive, reporting on as many of the common pathogens for a particular syndrome as possible. Sensitive and specific automated molecular diagnostic systems that detect up to 4 different pathogens in a single sample have been available from in vitro diagnostic (IVD) manufacturers for some time [27,28]. However, adoption of IVD platforms with broad multiplexing capability has become widespread only in the last few years. Commercially available systems that can detect most of the known etiological agents for respiratory, gastrointestinal (GI), and other multipathogen syndromes [29-31] include the BioFire (Salt Lake City, UT) FilmArray System ([32]; Multimedia Appendix 1); the GenMark (Carlsbad, CA) eSensor XT-8 [33] and ePlex [34]; and the Luminex (Austin, TX) xTAG [35], nxTag [36], and Verigene systems [37].

### Sharing of Patient Data

Multianalyte diagnostic tests provide the raw data needed for real-time pathogen-specific syndromic surveillance, but there remain a number of obstacles to sharing these results (reviewed in [38]). The obstacles largely center on information privacy and network security. A real-time surveillance system using diagnostic test results requires safeguards for protected health information (PHI). Medical records and devices have become attractive targets for cyber attackers in recent years [39], which has made hospitals and clinics reluctant to connect their local area networks (LANs) to the internet. Releasing patient test results requires the removal of PHI or authorization from the patient. Studies have shown that deidentification of patient data is not as simple as removing all specific identifiers because in the age of big data, under the right circumstances, it is possible to reassociate patients and their data using publicly available information [40-43].

We describe here the implementation of a real-time pathogen-specific surveillance system that overcomes the PHI concerns noted above. BioFire Syndromic Trends deidentifies, aggregates, and exports test results from FilmArray Instruments in use in US clinical laboratories [44]. Although data from all commercially available FilmArray panels [45] are exported to the Trend database, we focus here on the Respiratory Panel (RP) that can detect 17 viral (adenovirus, Adeno; coronavirus, CoV [OC43, 229E, NL63, HKU-1]; human metapneumovirus, hMPV; human rhinovirus/enterovirus, HRV/EV; influenza A, Flu A [subtyping H1N1, 2009 H1N1, H3N2]; influenza B, Flu B; parainfluenza viruses, PIVs [1-4]; and respiratory syncytial virus, RSV) and three bacterial (*Bordetella pertussis, Chlamydia pneumoniae, and Mycoplasma pneumoniae*) pathogens [32,46,47].

With more than 362,000 patient results for the FilmArray RP test alone, the Trend database has many of the properties associated with big data as it applies to infectious disease [48]. After describing how the dataset can be cleaned of nonpatient tests, we make some observations on the seasonality of the different respiratory pathogens and the occurrence of codetection (more than one organism is detected in one test). Relatively little is known about rates of multiple concurrent respiratory infections and their overall impact on the health of the patient. Finally, we apply the ecological concept of species diversity [49] to observe a correlation between the abundance of each pathogen and the rate at which codetections (more than one positive result per test) occur in the tested population.

## Methods

### Origin of Syndromic Trends

**Figure 1:**
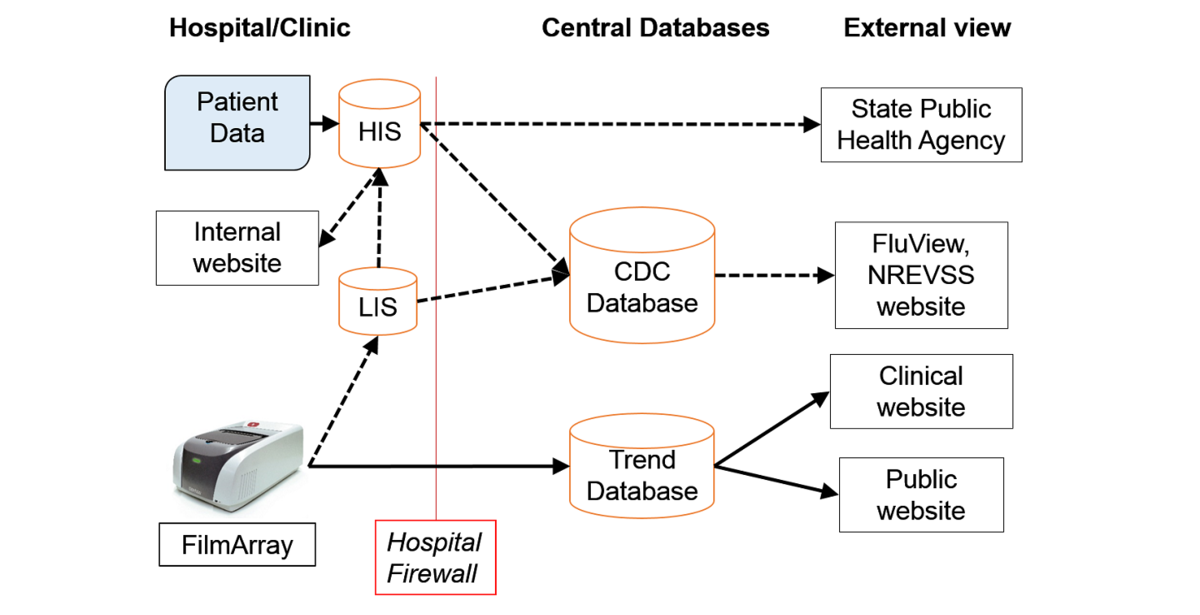
Schema for export of in vitro diagnostic (IVD) test results to an external database. Bottom-Out and Top-Out approaches for data export are indicated by solid and dashed lines, respectively. Some institutions have developed their own systems for aggregating and displaying infectious disease data (indicated by internal website). HIS: hospital information system; LIS: laboratory information system; CDC: Centers for Disease Control and Prevention; NREVSS: National Respiratory and Enteric Virus Surveillance Systems.

FilmArray Trend was originally implemented to provide BioFire customers with an up-to-date view of the respiratory and GI pathogens circulating at their institution. From the perspective of an IVD manufacturer, the most uniform and thus the simplest method of accomplishing this is to follow a bottom-out approach to data export in which the FilmArray sends data to a cloud database managed by the manufacturer, and Web views of these data are available by clinicians at the hospital that generated the data (solid lines in Figure 1) rather than a top-out approach (dashed lines in Figure 1) in which the data are extracted from the hospital information system. This method provides the clinical institution with a tool to perform pathogen-specific surveillance for very little cost.

### Patient Privacy When Exporting FilmArray Test Results

The Expert Determination study of the Trend data export algorithm (Multimedia Appendix 2) established that FilmArray patient results have been adequately deidentified. Therefore, a data use agreement (DUA), rather than business associates agreements (see Multimedia Appendix 2 for the difference between the two agreements) could be executed with each of the collaborating institutions (Multimedia Appendix 1). The DUAs define for the clinical laboratory how BioFire will manage and make use of the Trend data. The Trend client software residing on the FilmArray computer queries the FilmArray test result local database and exports the results to an Amazon Web Services database (Multimedia Appendix 1). The Trend client software performs deidentification on the FilmArray computer before export, as detailed in Multimedia Appendix 2. Health care providers (HCPs) are granted access to their institution’s Trend data by the laboratory director. As Web access to view the data is restricted to the local site, deidentification of geographic indicators is not required. However, in the implementation of the public Trend website, which presents FilmArray test results from around the United States, we have further aggregated the data with respect to geographic origin and obfuscated the date of the test (Multimedia Appendix 2). As only deidentified data are exported from the clinical institutions, no PHI is sent to or stored on the cloud server.

### Test Utilization Rate and Pathogen Detection Rate

**Figure 2:**
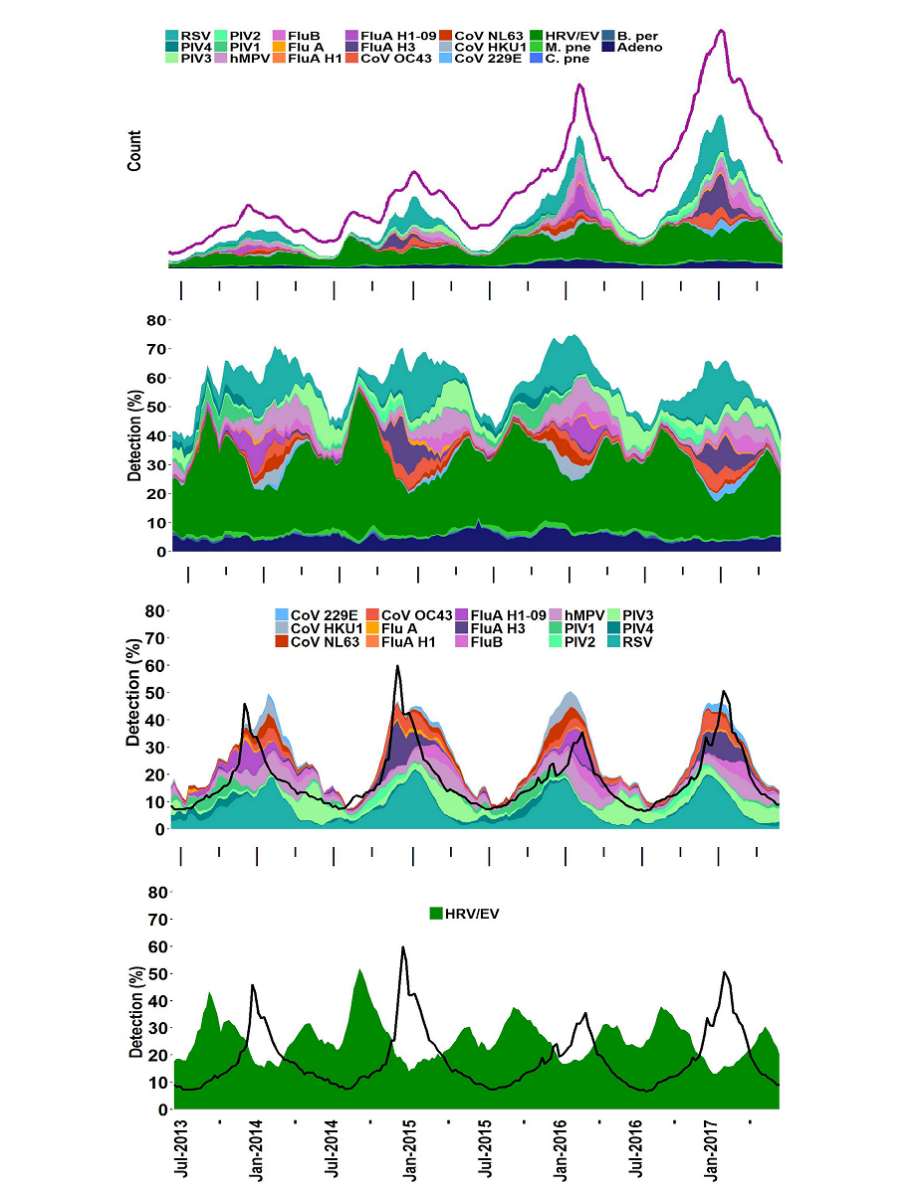
Detection of respiratory panel (RP) organisms over time across all sites. Detection of FilmArray RP pathogens in the Trend dataset displayed as stacked area graphs. All data views have the same time period (July 2013 through July 2017). (First data view) Count of each organism. The test utilization rate (TUR) metric (purple line, units are FilmArray RP tests performed) and count of FilmArray RP tests that are negative (white are between pathogen count and TUR) are indicated. The y-axis values are not indicated as this is considered proprietary information. (Second data view) Pathogen detection rates for all organisms. (Third data view) Pathogen detection rates for the subset of organisms that show seasonality (see Results and the legend for the list of organisms). (Fourth data view) Human rhinovirus (HRV) or enterovirus (EV) detection rates. The CDC weighted influenza-like illness (ILI; scaled up tenfold to be visible against the pathogen data) is indicated (black line) in the third and fourth data views. Organisms follow the same color scheme in all panels; the order of organisms in the legend (down then across) matches that of the stacked area graph top to bottom.

The FilmArray RP test utilization rate (TUR) metric is defined as the non-normalized number of RP patient test results generated each week across the Trend sites (computed as a centered 3-week moving average). To calculate the pathogen detection rate (as displayed in Figure 2 [second data view] and on the Trend website), we compute the rate for each organism at each institution as a centered 3-week moving average. To adjust for the capacity differences between sites, a national aggregate is calculated as the unweighted average of individual site rates. Only data from sites contributing more than 30 tests per week is included to avoid noise from small numbers of tests. Because the calculation of pathogen detection rate includes results from patients with multiple detections, the detection rate for all organisms can, in theory, add up to greater than one. In practice, this does not occur.

### Comparison With the Centers for Disease Control and Prevention Influenza-Observed Rate of Detection

The CDC FluView rate of Flu A and Flu B detections, as well as the reported incidence of weighted influenza-like illness (ILI), are taken from the CDC website [4]. Only the CDC data from the Department of Health and Human Services regions that contained Trend pilot sites (Multimedia Appendix 1) were used for calculating the rate of influenza detections.

### Calculation of Codetection Rates and Related Measures

Pathogen codetections are defined as FilmArray tests in which two or three organisms are detected. We also calculated two other measures that relate to codetections: the circulating pathogen number and the measure of interspecific encounter (MIE). Both of these time series measures are calculated for each site and week, a centered 5-week moving average is computed, and then an unweighted average of all sites is used to create a national aggregate. The 5-week moving average is used to reduce noise because of small numbers of samples within a week at some sites.

More specifically, the circulating pathogen number is simply the count of the unique organisms detected at a site during a 1-week period. MIE is calculated from the frequencies of each organism at a site (number of positive test results for an organism divided by the number of FilmArray tests performed at that site). To reduce noise, we only include site data if more than 10 FilmArray tests were performed in that week. If P_1_...P_N_ are the percentage detection of the N different organisms circulating at a single site over a single week, then MIE is defined as shown in equation 1:

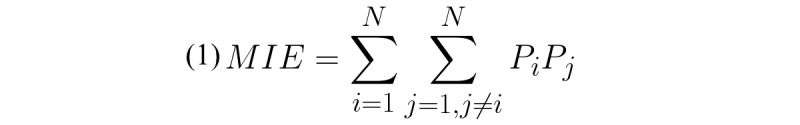



Conceptually, MIE is an attempt to estimate the likelihood that a patient infected with one organism may be infected with another unique organism circulating in the population at a given period in time, resulting in a coinfection.

## Results

### Sending FilmArray Data Directly to the Cloud

The most general and efficient way to aggregate test results from the FilmArray instrument in a clinical laboratory is to follow a bottom-out approach to data export (Figure 1; Multimedia Appendix 1). In this scheme, the FilmArray instrument (at the bottom of the information hierarchy) directly sends data via the internet to a single cloud database where it can be viewed by HCPs at the originating institution. This data export pathway contrasts with a top-out approach (Figure 1) in which diagnostic test results are pushed from the instrument up through the LIS, to the HIS (at the top of the information hierarchy) and, finally, a subset of this information is forwarded to cloud-based databases.

Initial testing of the Trend export mechanism was performed in collaboration with the clinical laboratories of the Medical University of South Carolina. This trial allowed us to develop and test auto-export functions and deidentification protocols for the Trend software. The deidentification requirement of the Health Insurance Portability and Accountability Act (HIPAA) of 1996, specifically the Safe Harbor provision, requires the removal of 18 enumerated variables that could directly or indirectly identify an individual [50]. In accord with this requirement, the first stage study did not export test identifiers or free-form text fields and only returned the year of the test. The initial dataset provided low-resolution information but was a useful platform to evaluate the proposed system. Further development to enable export of higher resolution data required the design of routines that would adhere to an alternative HIPAA deidentification strategy, namely, the Expert Determination approach, which requires a risk assessment demonstrating that the chance of reidentifying an individual is sufficiently small [51]. The Expert Determination process identified and made recommendations for fields that could facilitate disclosure of PHI (Multimedia Appendix 2). A summary of the Expert Determination results detailing the risk of Trend data in regard to replicability, availability, and distinguishability is shown in Multimedia Appendix 2.

All sites (Multimedia Appendix 1) submitted the Trend project for review by their local institutional review board; all but one of the 20 review boards deemed the project exempt because of the absence of PHI export. Thus, the security requirements for the database and the controls necessary for storage and transport of deidentified data are significantly reduced.

Following the protocol established by Expert Determination review, the Trend software delays the export of results until the number of tests queued for export exceeds a minimum threshold for each type of FilmArray panel. In practice, this results in an average time to export of less than 2 hours from each site that has multiple instruments. A total of 99.11% (74,912/75,585) of the test results exported automatically occurred within 24 hours of test completion.

### Characteristics of the FilmArray Sites Used in the Trend Pilot Study

The 20 sites contributing to the Trend pilot project (Multimedia Appendix 1) have the same average number of instruments; six (range: 1-22) as for all US FilmArray customers. The Trend pilot sites have been using the FilmArray RP test for an average of 3.8 years (range: 1-6) before June 2017. The size of the institutions participating ranges from 300 to 6400 beds, with the majority being large hospitals, and health care networks with an average of 1100 beds. Six (30%, 6/20) sites are pediatric hospitals, and one is a reference laboratory. Fifteen (75%, 15/20) of the sites have uploaded archived FilmArray RP test results to the Trend database, with eight (40%, 8/20) reporting results dating back to 2012. Unless stated otherwise, the data presented here cover the period from July 2013 to July 2017.

The algorithm used to diagnose the cause of respiratory disease varies by site. More than half of the Trend sites do not enforce an institutional respiratory testing protocol and, even within sites that have a required protocol, some discretionary use of FilmArray RP is allowed. Without detailed records from each institution’s HIS, it is not possible to determine whether the FilmArray RP was used as a front line test or as a reflex test (typically following a negative result for influenza and RSV).

### Cleaning Nonpatient Test Results From the Trend Database

To determine the prevalence of respiratory pathogens, we needed to expunge the Trend database of test results that are not derived from clinical patient samples. Nonpatient results come from a variety of sources including verification testing, routine quality control (QC), and proficiency testing (PT; Multimedia Appendix 3). Despite this complexity, the majority of nonpatient test results can be identified and distinguished from the patient-derived data because of the high number of positive organism calls in a single test and because of the temporal aspects of verification and control testing (Multimedia Appendix 3 shows one such identification method). QC tests are estimated to account for half of all FilmArray RP results in which more than three organisms are detected. In addition to the exclusion of tests temporally associated with validation events, all results with four or more positives were removed from further analysis (approximately 1% of the filtered total). This includes the small fraction of test results with exactly four organisms (Multimedia Appendix 3, Tests after event removal column) because the minority are derived from patient testing.

### Detection of Respiratory Pathogens in Trend Samples From 2013 to 2017

The detection counts and pathogen detection rates derived from the Trend dataset for each organism in the FilmArray RP are shown in Figure 2. Other views of these data, including percent detection of individual organisms or combinations of organisms, are available on the BioFire Syndromic Trends public website [44]. The FilmArray RP TUR (see Methods) and the individual organism detection counts increased over this period because the Trend clinical sites increased their utilization of the FilmArray RP tests (Figure 2, first data view). Seasonal fluctuations can also be seen within this growth pattern, with use increasing up to four-fold each winter when compared with the previous summer. HRV/EV, the most common pathogen detected group, is identified in approximately one-fourth of all samples tested each year (Multimedia Appendix 4). Other pathogens detected in approximately one-tenth of the samples include RSV, the PIVs, ADV, influenza, and hMPV. *M pneumoniae, C pneumonia,* and *B pertussis* are detected in a small percentage (one-fiftieth) of all samples. The average percentage of each organism is relatively constant over the 4 years of data in the Trend database (Multimedia Appendix 5).

The pathogens’ seasonal variability measured by percent detection can be classified into at least three groups. Group 1: the majority of organisms follow the classical respiratory season (October-March) and increase by more than ten-fold above their baseline detection rate (Figure 2, third data view). These include the CoVs, Flu A, Flu B, hMPV, the PIVs, and RSV (PIV3 is a slight exception to this rule in that it peaks in the summer months and has a winter peak that is only detected regionally; data not shown). Within this group, all but five viruses demonstrate significant fluctuations from year to year; Flu B, hMPV, OC43, and PIV3 and RSV experience relatively consistent annual peaks. Group 2: HRV/EV is in a class by itself in that it is detected in a high percentage of tests over time (minimum of one-tenth of tests in winter) and experiences moderate peaks of two- to three-fold outside the respiratory season baseline in the early fall and spring (Figure 2, fourth data view). Group 3: the bacteria and Adeno are present at a relatively constant rate (Multimedia Appendix 6). The CDC FluView reported rate of ILI tracks moderately well with the group 1 organisms (cross-correlation of 0.85) and not with HRV/EV or with Adeno and the bacteria.

### Comparison of Trend With Centers for Disease Control and Prevention Measures of Influenza

**Figure 3:**
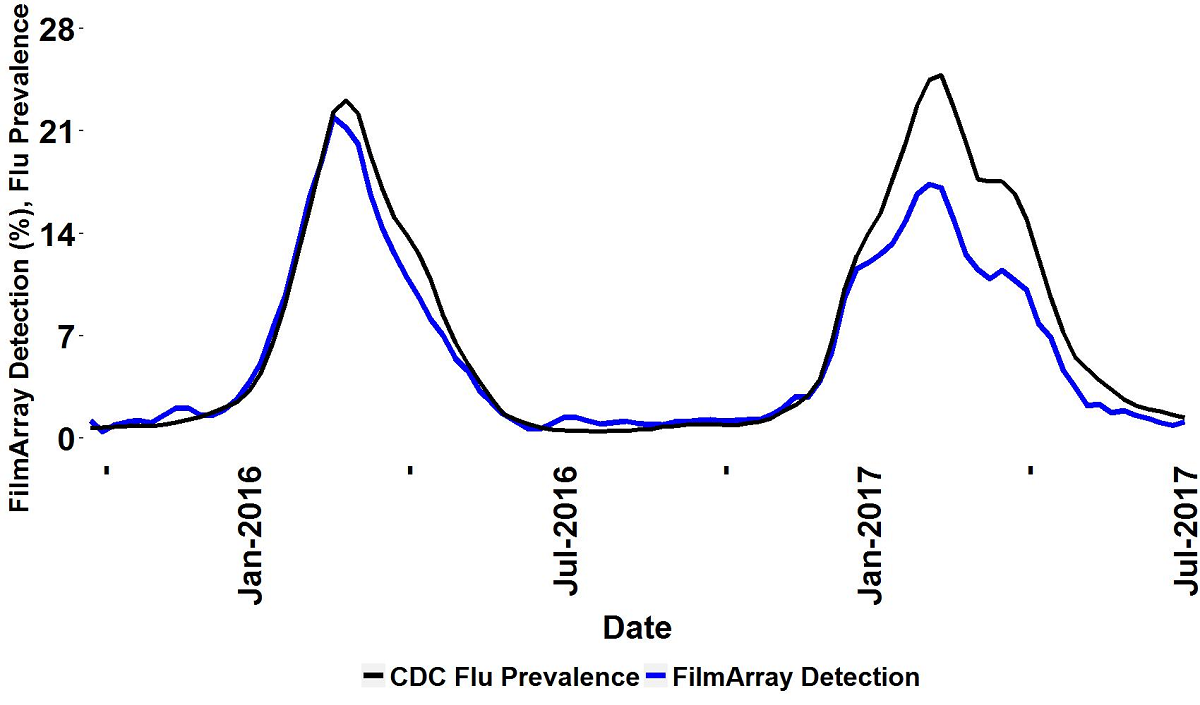
Trend influenza detection rate compared with Centers for Disease and Prevention’s (CDC) influenza activity. Percent of combined FilmArray Flu A (all subtypes) and Flu B detections (blue line) and CDC-reported influenza prevalence (black lines). CDC data are aggregated only from regions with participating Trend sites.

The CDC FluView network [4] gathers information about influenza prevalence from a large number of public health and clinical laboratories in the United States. FluView is considered the gold standard for these measures. We compared the Trend detection rates for Flu A (all subtypes) plus Flu B with the FluView Influenza (A and B) from September 2015 to July 2017 (Figure 3). The analysis was restricted to this time period because of a change in the CDC’s reporting of flu prevalence in the fall of 2015. A cross-correlation of 0.974 was observed between the Trend Flu A or B percent detection and FluView reported influenza prevalence. Notably, the onset, peak, and duration of the influenza season coincide between the two measures.

### Respiratory Panel Codetections

**Figure 4:**
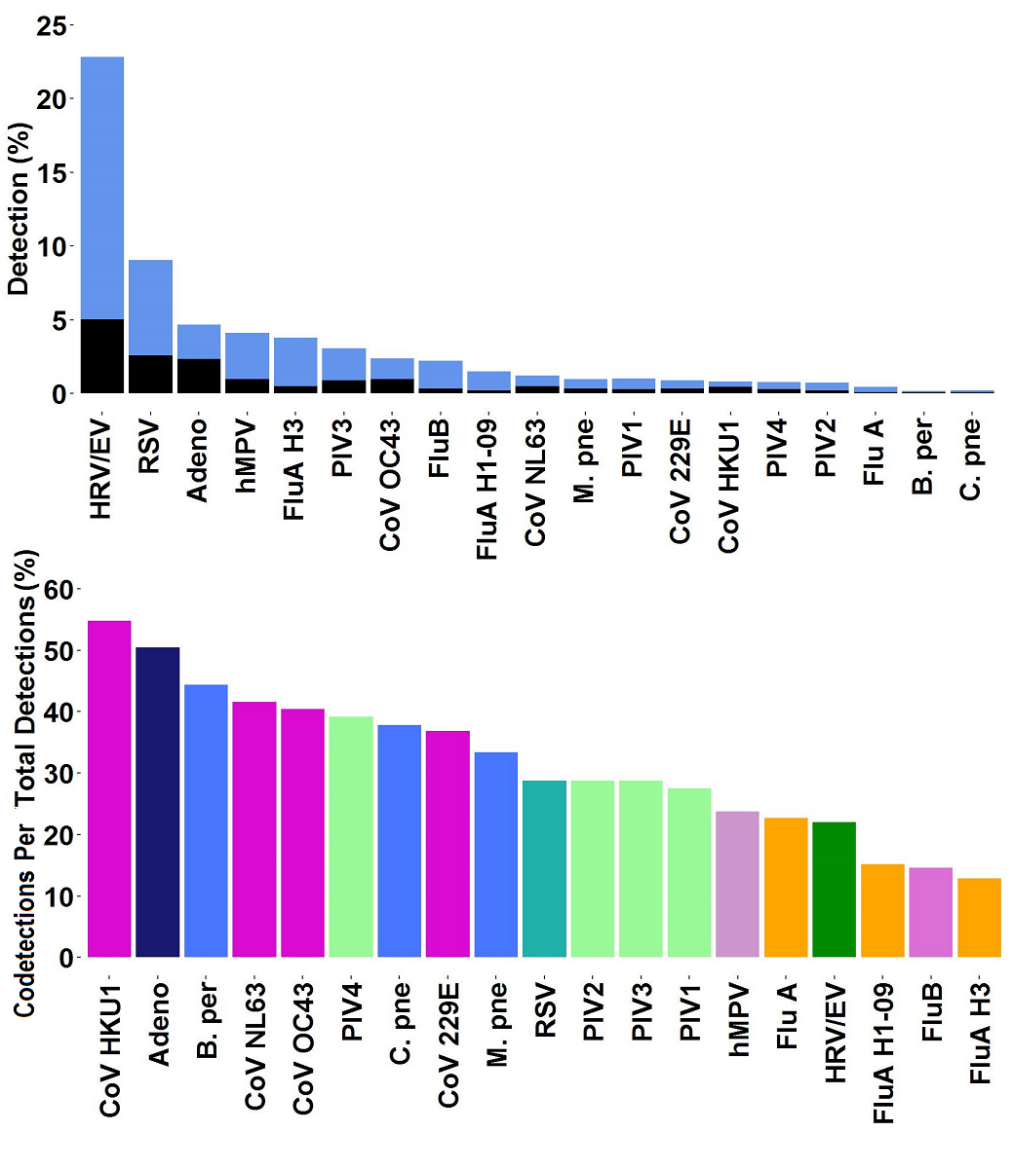
Detection rates for all organisms compared with codetections. Percent total positive detections for each organism in the respiratory panel (RP) Trend dataset is presented in stacked bars, showing the rate of detection of a single organism (first data view, blue) and those involved in a codetection (first data view, black). Data are calculated for each site during the period from July 2013 to July 2017, when available, and then aggregated. (Second data view) Percentage of each organism involved in a codetection is shown. Bars are colored by pathogen family (CoV, purple; bacteria, blue; PIVs, green; Flu A, yellow).

We found that approximately 38,000 FilmArray RP tests in the Trend dataset had two or three codetections. The most common codetections observed are those involving HRV/EV, which is the pathogen with the overall highest rate of detections (Figure 4, first data view). The codetection rate within each organism varies widely (from one-tenth to one-half; Figure 4, second data view). Although an additional pathogen was detected in half of the Adeno and CoV positive samples, codetections were observed in only one-tenth of the samples positive for either Flu A or Flu B (Figure 4, second data view).

**Figure 5:**
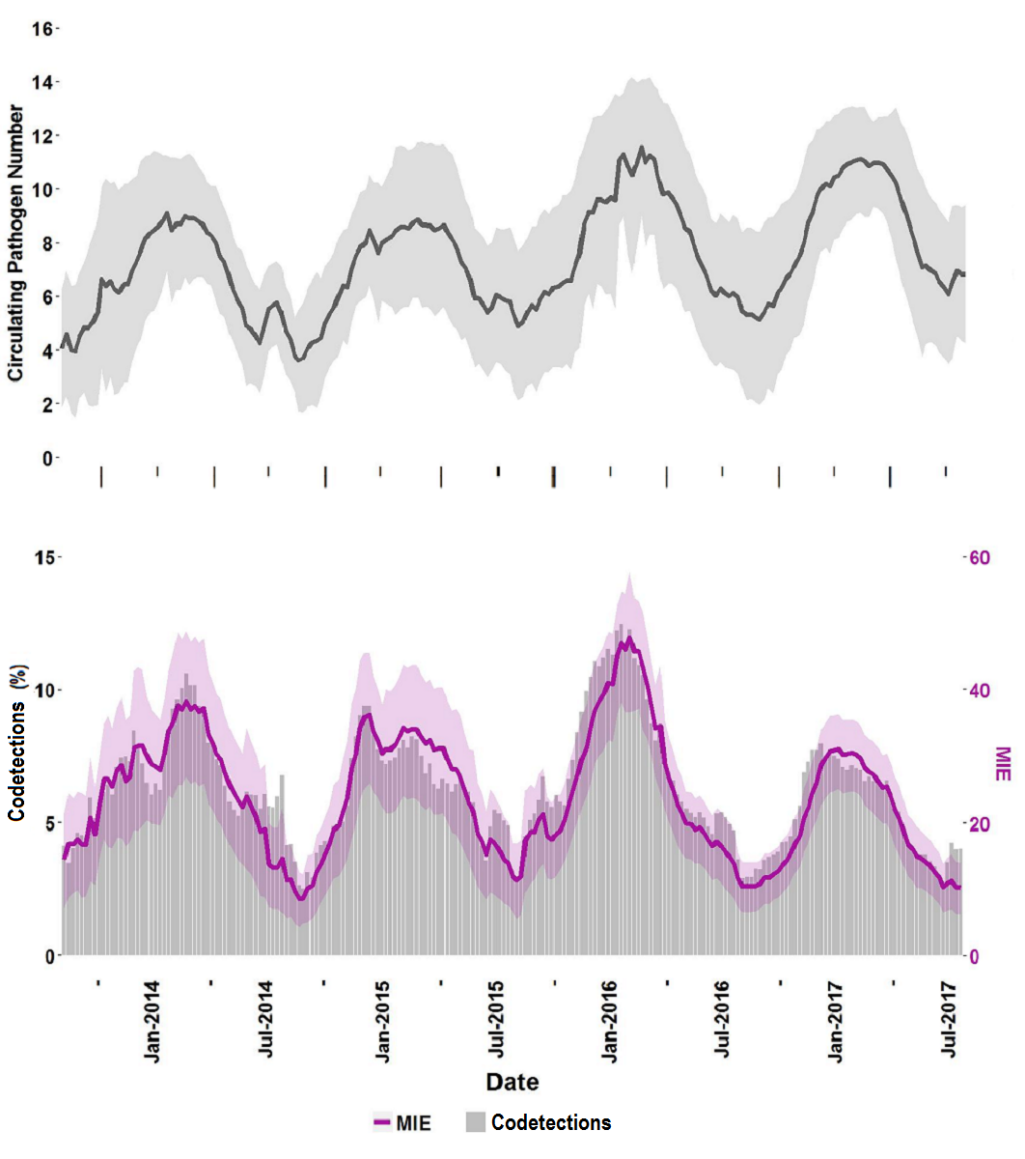
Seasonal variation in pathogen diversity and codetections. (First data view) Average circulating pathogen number (black line) and one SD computed across all Trend sites (gray area). (Second data view) Rate of codetections in the respiratory panel (RP) Trend dataset (gray bars, left axis), the measure of interspecific encounter (MIE) index (purple line, right axis), and MIE CIs (shaded purple area).

Trend data have high temporal, spatial, and organism-specific resolution. These three properties allow for a novel evaluation of codetections. The observed rates of codetections should be influenced by the number of circulating pathogens detected by the FilmArray RP test at a particular site. Figure 5, first data view, shows the average number of unique organisms detected at each site in a given week (see Methods: Calculation of codetection rates). This number fluctuates from a summer low of four to a winter high of 11 pathogens. Figure 5, second data view (gray bars), shows that the total rate of organism codetections in the Trend dataset fluctuates annually, with peak rates occurring in the winter months. The average rates have been as high as one in 8 tests in the winter of 2016 and as low as one in 50 in the summer of 2014.

From the Trend data, an MIE can be calculated as the probability of a codetection, weighted by the prevalence of each circulating pathogen at a site. Although the value of the MIE metric is higher than the actual codetection rate, it correlates well (Figure 5, second data view, purple line compared with the gray bars has a cross-correlation of 0.9488 at a lag of 0). The magnitude adjustment between MIE and the observed codetections is calculated by the slope of the linear regression of the two metrics (Multimedia Appendix 7) and has a value of 4.05 (*R*^2^=.9003).

## Discussion

### Properties of Trend Data

This study describes BioFire Syndromic Trends, a new system for real-time reporting of widespread pathogen-specific syndromic data. Even in its pilot phase, the Trend database already has many of the features that characterize big data [48]. The Vs of big data—volume (amount), velocity (speed of acquisition), veracity (accuracy), variety (diversity of information), and value (utility)—should be kept in mind as we consider the properties of Trend in clinical and public health settings.

The Trend RP dataset is growing at an average rate of >400,000 pathogen test results per month (>20,000 patient tests with 20 pathogens). Connecting the first 20 clinical sites has provided insight into the principal concerns that will be raised by the legal, information technology, and administrative departments of the HCPs that house FilmArray instruments. It should be possible, therefore, to expand the Trend installed base by 10- to 20-fold over the next few years. Similarly, the existence of Trend should enable other IVD manufacturers to build their own Trend-like systems with greater acceptance on the part of their customers, thereby allowing a more global and comprehensive surveillance perspective.

The data in Figure 2 are similar to previous demonstrations of the seasonality associated with different respiratory viruses [52-55]. What is novel is that these data are generated automatically, on site, and in close to real time compared with other surveillance systems. Nearly all of the test results are exported to the Trend database within 24 hours of being generated. As part of the deidentification protocol, sequential FilmArray RP tests of the same type are put into the same time bin. This has the effect that test results are exported faster during periods of peak use, such as during the peak of the respiratory season or during an outbreak. Trend should be instrumental at a local level to determine the start of a respiratory season; many hospitals make significant changes to their operations based on this event; however, at present, data collection to track the respiratory season is often slow and manual, or semiautomated at best.

The key to implementing Trend clinical sites was to demonstrate that FilmArray test results can be exported without the risk of breaching PHI confidentiality either directly or through some combination of the data that were exported. Trend successfully used the Expert Determination process as prescribed by the HIPAA guidelines (see Multimedia Appendix 2), which greatly simplified the data sharing agreement between BioFire Diagnostics and the clinical site and allowed HCPs to use Trend without risk of inadvertently disclosing PHI.

The software architecture underlying the Trend system is both simple and secure: (1) no changes to the institutional firewall or LAN are needed; (2) the Trend database cannot reach back and query the FilmArray computer because of the institutional firewall, which is set to outbound data only; and (3) Trend software can only submit data to the cloud database and cannot query the database (Multimedia Appendix 1). Yet, despite this security, authorized users of the Trend database can mine the deidentified data to look for novel patterns in respiratory pathogen epidemiology.

### The Costs and Benefits of Bottom-Out Data Export System

The goal of an epidemiological surveillance network is to infer which infectious diseases are circulating in the general population based on testing a sample of patients [56]. Different surveillance systems have different biases in their data; biases that perturb the ability to predict true population prevalence.

Although the removal of all PHI has great benefits in terms of implementation, it also has several shortcomings that complicate interpretation of the data. First, Trend cannot account for the variability in the diagnostic testing algorithms applied to the selection of samples to be tested by the FilmArray instruments. During the respiratory season, HCPs may prescreen patients with other diagnostic tests including rapid antigen or molecular assays for influenza and RSV or commercial and laboratory-developed molecular tests for a mix of other respiratory pathogens. Depending upon the sensitivity of these upstream tests, more than half of influenza and RSV for the subset of the patients screened would be excluded from the Trend dataset if the front line test is positive. This testing protocol may skew the actual prevalence of not only influenza and RSV but all other individual respiratory pathogens and coinfections detected by the FilmArray. In some institutions, testing is reserved for hospitalized patients and others at risk for developing complications of respiratory tract infections, including the very young, very old, and immunocompromised patients. So Trend data may represent a less healthy patient population and not necessarily general community prevalence. Conversely, there are sites that perform a significant number of tests for the outpatient setting. This may create variability among the clinical sites’ percent positivity and introduces a challenge to comparing pathogen intensity between sites.

The uncertainties surrounding the testing algorithm and the precise patient population tested should not interfere with determining the onset, peak, and duration of the pathogen season at each institution. These limitations on the data are likely to be common among almost all current surveillance systems for similar reasons. Given these concerns, the agreement between the percent positivity of Flu A or B as determined by Trend and the percent positivity reported by CDC FluView Influenza is striking (Figure 3), supporting the validity and utility of the Trend data.

The second source of concern in the Trend dataset is a consequence of the removal of sample identification such that we cannot directly determine whether the sample was from a patient or was a nonclinical sample (verification test, QC, or PT) and should be removed from further epidemiological analysis. We estimate that nonpatient testing makes up approximately one-fiftieth of the total FilmArray RP tests. Automated detection algorithms remove roughly one in 25 of the total RP tests, including approximately half of the nonclinical samples. With the exception of the four positive tests, the clinical samples removed by filtering should be a random sampling of all patient tests. The remaining nominal fraction of nonpatient tests has essentially no impact on the Trend evaluation of pathogen prevalence, but they do make it more difficult to perform high-resolution analysis of pathogen codetections. This is especially true for codetections of low prevalence organisms where QC positives are likely to be more common than real positives. Future updates to the FilmArray software will simplify the process by which the instrument operator can tag tests of nonpatient samples, thereby largely eliminating the need to filter such test results from the Trend database before analysis.

### The Seasonality and Coinfections of Respiratory Pathogens

The total positivity rate of the FilmArray RP test varies from a low of approximately one-third of tests in the summer months to a high of three-fourths of the tests in December and January. Figure 5, second data view, shows that the average number of different circulating pathogens at a single institution can vary from eight up to 11 during the winter months. Even during the peak periods of ILI, many respiratory infections are due to other viruses (Figure 2, third data view) that can present clinically in a similar fashion [57,58]. Therefore, the presumption of an influenza infection based on reported influenza percent positivity, without diagnostic testing for the virus, can lead to the inappropriate use of antiviral agents [59]. Conversely, without comprehensive testing, a negative influenza or RSV test can lead to the prescription of an unnecessary antibiotic. Trend data can be a valuable aid for antimicrobial stewardship programs because it provides real-time information regarding the causes of respiratory infections and highlights the prevalence of viral infections.

As previously observed [55], the viruses that share the winter seasonality of influenza demonstrate annual or biennial behavior. It is possible that the viruses that share an influenza-like seasonality but do not show a two-year cycle (RSV and hMPV) are actually alternating strains, but the FilmArray RP Test does not detect this difference (eg, the FilmArray RP does not differentiate between RSV A and RSV B). Adeno and the bacteria show constant occurrence through the year; HRV is in a unique class with peaks in the fall and spring.

Detection of multiple respiratory viruses in the same patient has been reported before. In the Trend dataset, the rate of dual and triple codetections was approximately 7.94% (28,741/362,101), with HRV/EV as the organism most commonly observed in a codetection. Some viruses such as ADVs and the CoVs are detected in the presence of another organism approximately half of the time (Figure 4). In principle, a FilmArray RP positive result may represent detection of residual pathogen nucleic acid from a previous infection that has resolved. However, several studies suggest that coinfections are associated with more severe disease [60-62] (see also discussion in [63]). In such cases, information about multiple detections can provide infection control practitioners with data that can assist in bed management and in the assessment of risk for nosocomial infections in a patient population that has been segregated by the occurrence of a common pathogen. Such information can prevent the introduction of a new pathogen associated with cohorting patients during busy respiratory seasons [64-66].

The question of whether different respiratory pathogens interfere with, or facilitate, growth in a human host is of some interest and not well understood. With the right data, it can be studied at the population [67], individual [68], and cellular level [63]. Because the Trend data still include some nonpatient tests, we have chosen not to analyze every possible dual or triple infection individually. Rather, we have taken a global approach and compared the overall rate of observed codetections with MIE, which is a measure of the diversity of viruses circulating in a specific region and time period. MIE is similar, but not identical, to Probability of Interspecific Encounter (PIE [69]), also referred to as the Gini-Simpson index (1-D, where D is the Simpson’s index), which is used in ecology as a measure of the species diversity of a region. Similarly, the circulating pathogen number of Figure 5, first data view, is identical to the Species Richness measure of ecology. We calculate MIE using frequencies (P_i_) of pathogen positivity per FilmArray test and note that the sum of all pathogen frequencies can add up to more than unity because of codetections or be less than unity because of the presence of negative tests. In this regard, MIE differs from PIE because it is not a probability measure.

Figure 5, second data view, shows that the observed rate of codetections is a constant fraction of MIE (approximately one-quarter as indicated by the linear regression of Multimedia Appendix 7). This observation suggests that, in the aggregate, respiratory pathogens are appearing in coinfections at a rate that can be predicted by their observed abundance. The data, however, may be biased by the patient population tested and the type of respiratory disease. The data also does not rule out that there are particular respiratory pathogens that occur more or less often in mixed infections than predicted by their individual percent positivity rates [63,70]. As we improve our ability to remove nonpatient test results from the Trend dataset, we will be able to characterize specific virus codetection rates and their significance [54,55,67,68,71,72].

### Applications of Trend Data

As with weather forecasting, there is both a theoretical and a practical interest in predicting the next few weeks or months of the respiratory season [73-76]. Trend contributes to infectious disease forecasting efforts because the data are timely and comprehensive. As the number of sites participating in Trend increases, it will be possible to localize the reported infections to smaller geographical regions. At a high enough density of Trend sites, patterns of movement of respiratory pathogens across the United States will become visible in a way that has not been easily observed before now.

The Trend RP data show the percentage contribution of each pathogen to what is currently being detected by FilmArray RP testing (Figure 2, second data view) [44]. This analysis does not take into account changes in the rate of testing over a given season; information that should provide additional data regarding disease intensity and severity. In contrast, the simple metric, TUR, describes the non-normalized rate of FilmArray test usage and serves as a surrogate for the level of syndromic disease that HCPs observe (Figure 2, first data view).

TUR suffers from two defects. First, it is closely linked to the sales of the FilmArray test and thus is proprietary data that BioFire does not share (Google took a similar position in regard to releasing the search queries used by Google Flu Trends [12]). Second, TUR is driven by both the demand for testing and the growth in FilmArray product adoption and increasing acceptance and usage by HCPs. A useful step beyond TUR would be a normalized rate that can adjust for the underlying growth of testing unrelated to the intensity and duration of the respiratory disease season. An increase in a normalized TUR metric may indicate the prevalence of circulating respiratory viruses and the intensity of respiratory disease overall. Likewise, an increase in the normalized metric, concomitant with an increase in negative tests, may indicate the occurrence of an outbreak caused by an emerging pathogen.

Public health agencies, which include local and state health departments and the CDC, are specifically exempt under a HIPAA provision that allows clinical laboratories to disclose PHI to the agencies for specified public health purposes [77]. The exemption includes follow-up studies on reportable infectious diseases. Real-time pathogen-specific syndromic surveillance systems such as Trend will allow state health departments to more rapidly identify, acquire, and test residual samples from potential outbreaks. Conversely, perceived outbreaks may actually be coincidental multi-organism seasonal surges, and rapid analysis by Trend-like systems could prevent timely and costly outbreak investigation.

Given the movement in health care technology toward greater vertical integration of a hospital’s data, the bottom-out approach exemplified by Trend will face more competition from top-out approaches (Figure 1, see, eg, GermWatch in Utah, [21]) because these systems can capture patient information (eg, age, gender, and patient address) that is critical for more detailed epidemiological analysis. However, combining PHI with the diagnostic test result in the top-out approach makes these systems more complex and difficult to implement and may limit participation by health care institutions. Ironically, bottom-out data export systems have a role to play in the development of top-out systems because bottom-out export provides a rapid and efficient means to quality check the data flowing from top-out systems. Trend data could also be combined with data derived from other automated diagnostic platforms [78,79]. This work might best be accomplished by a third party that is viewed as independent and impartial. For example, in the case of data originating in the United States, a federal institution or a private foundation could host a database to which IVD manufacturers would contribute their different syndromic test results. The benefits of a more complete view of circulating pathogens should outweigh the complexities of combining data from different platforms.

### Future Outlook

Syndromic Trends is a novel surveillance tool for simultaneously monitoring multiple syndromic diseases that has demonstrated promise in expanding our knowledge of the epidemiology of infectious diseases. Indeed, the close correlation of seasonal respiratory viruses tracked by Trend with reported CDC ILI highlights the major contributory role of multiple respiratory pathogens beyond influenza to ILI. The national and global expansion of Trend will provide a comprehensive tool to study the impact of coinfections, understand the role of previously underappreciated pathogens, and clarify true disease epidemiology. Finally, systems such as Trend will be essential for the rapid identification of disease anomalies indicating potential emergent outbreaks, thereby providing an independent tool for public health surveillance.

## Appendix

Multimedia Appendix 1 BioFire Syndromic Trends System.

Multimedia Appendix 2 Deidentification of Patient data for Infectious Disease Epidemiology.

Multimedia Appendix 3 Cleaning Trend Data.

Multimedia Appendix 4 Detection of FilmArray RP Organisms by Type.

Multimedia Appendix 5 Detection of FilmArray RP Organisms by Year.

Multimedia Appendix 6 Detection of Adenovirus and the Three Bacteria.

Multimedia Appendix 7 Linear Regression of MIE and Observed Codetections.

## Abbreviations

Adeno: adenovirus
CDC: Centers for Disease Control and Prevention
CoV: coronaviruses
DUA: data use agreements
ECLRS: Electronic Clinical Laboratory Reporting System
Flu: influenza
GEIS: Global Emerging Infections Surveillance
HCP: health care provider
HIPAA: Health Insurance Portability and Accountability Act
HIS: hospital information system
hMPV: human metapneumovirus
HRV/EV: human rhinovirus/enterovirus
ILI: influenza-like illness
IVD: in vitro diagnostic
LAN: local area network
LIS: laboratory information system
MIE: measure of interspecific encounter
NREVSS: National Respiratory and Enteric Virus Surveillance Systems
PHI: protected health information
PIE: probability of interspecific encounter
PIV: parainfluenza virus
PT: proficiency testing
QC: quality control
RP: respiratory panel
RSV: respiratory syncytial virus
TUR: test utilization rate
