# Supplementary material for: Automated Real-Time Collection of Pathogen-Specific Diagnostic Data: Syndromic Infectious Disease Epidemiology
Source: JMIR Public Health Surveill. 2018 Jul 6;4(3):e59. doi: 10.2196/publichealth.9876 (PMC6054708; doi:10.2196/publichealth.9876)
Supplement: Multimedia Appendix 1 [file publichealth_v4i3e59_app1.pdf]

Multimedia Appendix 1: BioFire Syndromic Trends System

System and Data Output

The BioFire® FilmArray® System performs nucleic acid purification, reverse transcription, nested multiplex Polymerase Chain Reaction amplification and DNA melt curve analysis for up to 30 targets in 63 minutes ([32], www.biofiredx.com). The FilmArray disposable pouch is prepared by injecting hydration solution and, separately, the patient sample, i.e., for the FilmArray RP test, a nasopharyngeal swab in viral transport media. The pouch is loaded into the FilmArray Instrument and the operator scans the barcode (containing panel type, lot and serial numbers) and then scans or manually enters information into the "Sample" field. Additional free form text can be entered into a "Tag" field. Software on the computer connected to the FilmArray Instrument analyses the amplification products and indicates each pathogen target detected. The results are presented as a Portable Document Format report and can be exported to the laboratory's LIS. Information about the patient (gender, age, date of birth etc.) is not recorded by the instrument unless specifically entered.

As of 2016, more than 4,000 FilmArray instruments have been placed in clinical use worldwide. Approximately 80% of these systems are in the US. The FilmArray RP test is United States Food and Drug Administration designated as Clinical Laboratory Improvement Act (CLIA) moderate status (can be used in moderate to high complexity laboratories).

Syndromic Trends Client Software and Database

The Trend client software resides on the computer associated with the FilmArray Instrument(s). The computer makes a secure, Hypertext Transfer Protocol Secure connection to services hosted by Amazon Web Services. Authenticated data submissions are stored in a database hosted by Amazon Relational Database Service. Both services have been configured to be HIPAA Security Rule compliant [80]. Trend client software requires that the computer has Internet access to make secure outbound web requests. The connection protocol is industry standard technology used for secure banking and web applications. The Website software authenticates the Trend data before saving the exported de-identified data to the Trend Database.

Multimedia Appendix 1 Table: Clinical laboratories participating in the initial Trend study

HHS region[a]	Name	City, State	Hospital, Facility Type	Bed Count
1	University of Massachusetts Medical School-Baystate	Springfield, MA	University Affiliated	1,000
2	Albany Medical Center	Albany, NY	Medical College Affiliated	800
2	Northwell Health	Lake Success, NY	Network	6,400
2	NYU Langone Health	New York, NY	Network	1,500
2	Stony Brook University Hospital	Stony Brook, NY	University Affiliated	600
2	Winthrop University Hospital	Mineola, NY	University Affiliated	600
4	Greenville Health System	Greenville, SC	Network	1,400
4	Nicklaus Children's Hospital	Miami, FL	Children's	300
4	Medical University of South Carolina	Charleston, SC	University Affiliated	700
5	South Bend Medical Foundation	South Bend, IN	Commercial lab serving regional hospitals	NA
5	Detroit Medical Center	Detroit, MI	Network	2,000
5	Nationwide Children's Hospital	Columbus, OH	Children's	600
7	Children's Mercy	Kansas City, MO	Children's	400
7	Washington University Medical Center	St. Louis, MO	University Network	1,700
7	Nebraska Medical Center	Omaha, NE	University Network	600
8	Children's Hospital Colorado	Denver, CO	Children's Hospital	500
8	Intermountain Medical Center	Salt Lake City, UT	University Affiliated	500
8	Primary Children's Hospital	Salt Lake City, UT	Children's	300
9	Children's Hospital of Los Angeles	Los Angeles, CA	Children's, University Affiliated	500
9	UC San Diego Health	San Diego, CA	University Affiliated	700

[a] The Health and Human Services (HHS) regions are as defined by the CDC (reference [4]).

References

4. National Center for Immunization and Respiratory Diseases. National Respiratory and Enteric Virus Surveillance System (NREVSS). Centers for Disease Control and Prevention; 2017 [April 30, 2017] Available from: http://www.cdc.gov/surveillance/nrevss/. Archived at: http://www.webcitation.org/6wY28o3ZP.

32. Poritz MA, Blaschke AJ, Byington CL, Meyers L, Nilsson K, Jones DE, Thatcher SA, Robbins T, Lingenfelter B, Amiott E, Herbener A, Daly J, Dobrowolski SF, Teng DH, Ririe KM. FilmArray, an automated nested multiplex PCR system for multi-pathogen detection: development and application to respiratory tract infection. PLoS One 2011; 6(10):e26047

80. Amazon Web Services. Architecting for HIPAA Security and Compliance on Amazon Web Services. Amazon; 2017 [April 30, 2017] Available from: https://d0.awsstatic.com/whitepapers/compliance/AWS_HIPAA_Compliance_Whitepaper.pdf. Archived at: http://www.webcitation.org/6wY36JCmV.
