# Supplementary material for: Automated Real-Time Collection of Pathogen-Specific Diagnostic Data: Syndromic Infectious Disease Epidemiology
Source: JMIR Public Health Surveill. 2018 Jul 6;4(3):e59. doi: 10.2196/publichealth.9876 (PMC6054708; doi:10.2196/publichealth.9876)
Supplement: Multimedia Appendix 2 [file publichealth_v4i3e59_app2.pdf]

Multimedia Appendix 2: De-identification of patient data for infectious disease epidemiology

HIPAA Requirements for De-identification of Patient Information.

The Health Insurance Portability and Accountability Act of 1996 (HIPAA) and the Health Information Technology for Economic and Clinical Health Act (a part of the American Recovery and Reinvestment Act of 2009) apply to the use of IVD test results. The US Department of Health and Human Services (HHS) has issued regulations that set standards for the use of patient data. Under HIPAA, laboratories that perform the BioFire® FilmArray test on patient samples are "Covered Entities". If laboratories send PHI to a third party (such as the IVD manufacturer) then that party is acting as a business associate (BA) of the Covered Entity and a Business Associate Agreement would be required.

The HIPAA Privacy Rule [81] dictates that Covered Entities and, by extension, their BAs, may only disclose PHI with the patient's written authorization for the purposes of treatment, payment or normal health care operations, and a small number of additional exemptions [77]. Trend software resolves HIPAA concerns by removing all PHI before the test results leave the Covered Entity. This makes it highly unlikely that BioFire, or a malicious intruder into the Trend database, could associate the FilmArray test results with a patient. Because PHI is not exported, BioFire is not a BA and a Data Use Agreement (DUA) between the laboratory and BioFire addresses the export of FilmArray data to the Trend database. Demonstrating that Trend database does not contain PHI has been critical to recruiting institutions to this project.

Expert Determination of De-identification

The HIPAA Privacy Rule allows for release of patient data without prior authorization as long as it has been properly de-identified [50]. There are two acceptable routes to de-identification, 1) the Safe Harbor approach wherein the data are stripped of an enumerated list of 18 identifiers classified as PHI and there is no indication (i.e., actual knowledge), that the remaining information would lead back to the individual and 2) the Expert Determination approach wherein a person with experience in relevant statistical and scientific principles evaluates the PHI in conjunction with other reasonably available records, establishes a protocol for de-identification and certifies that the protocol allows only a small risk that PHI may be disclosed to an anticipated recipient.

The goal of the Trend project is to provide a near real-time view of the changes in pathogen prevalence; therefore, it is important to be able to retrieve the date when a FilmArray test is performed. However, retrieving the date conflicts with the Safe Harbor approach because the date of a test is PHI (the year of the test is not PHI but working with just the year defeats the purpose of tracking prevalence through the season). For this reason we followed the Expert Determination approach to manage data export.

The study took into consideration data that are available on participating clinical laboratory FilmArray Instruments, BioFire's own customer database, the proposed Trend database and publicly available data sources. We analyzed how combinations of this information could be used by an adversary to identify an individual in the dataset thereby disclosing PHI [82]. The results of this study (summarized in Multimedia Appendix 2 Table) provided recommendations for development and site enrollment criteria for Trend, and for BioFire operating procedures.

In accord with the recommended actions, information in the fields that may be used to distinguish a patient is obfuscated (through truncation or binning) to ensure that a combination of these fields cannot be used to identify a specific patient [50]. For example, the time and date of the test are dynamically binned so that a minimum number of tests of one panel type are included in each bin prior to export. This ensures that a sufficient quantity of test results are uploaded to the database from one site at one time so that there is very low risk that patient identity can be inferred from knowledge of the start time of the test.

If an adversary were to infiltrate the safeguards of the database, and wished to know specific patient test results from a specific location on a given day, no unique records would exist. The combination of deleting the sample identification field, binning the test date range, and truncating the FilmArray pouch serial number ensures that the remaining information is never unique, which indicates that there is a low risk of misuse of data.

Multimedia Appendix 2 Table: Qualitative analysis for the Expert Determination

Field	Replicability	Availability	Action
Sample	**High** (continually associated with a patient)	**High** (name exits in public records)	Deleted, the Trend database is not configured for the capture of this field.
Test Time and Date	**Medium** (only associated with patient once)	**Medium** (may be self-disclosed in public)	Binned into date range when minimum threshold of individuals is met
FilmArray Pouch Serial Number	**Medium** (only associated with patient once)	**Low** (only available to institution)	Truncated<br>(last digit removed)
Test Location	**High** (likely continually associated with a patient)	**Medium** (may be self-disclosed in public)	Limited to sites drawing from a minimum population (e.g., >20,000 individuals by census).
Instrument Name	**Low** (not associated with patient)	**Low** (only available to institution)	No Action

References:

50. Office for Civil Rights. Guidance Regarding Methods for De-identification of Protected Health Information in Accordance with the Health Insurance Portability and Accountability Act (HIPAA) Privacy Rule. U.S. Department of Health & Human Services; 2012 [April 30, 2017] Available from: https://www.hhs.gov/sites/default/files/ocr/privacy/hipaa/understanding/coveredentities/De-identification/hhs_deid_guidance.pdf. Archived at: http://www.webcitation.org/6wY2qwlc.

77. Office for Civil Rights. Disclosures For Public Health Activities. U.S. Department of Health & Human Services; 2003 [April 30, 2017] Available from: https://www.hhs.gov/sites/default/files/ocr/privacy/hipaa/understanding/special/publichealth/publichealth.pdf. Archived at: http://www.webcitation.org/6wY2zvbOH.

81. Office for Civil Rights. Summary of the HIPAA Privacy Rule. U.S. Department of Health & Human Services; 2003 [September 20, 2017] Available from: https://www.hhs.gov/sites/default/files/privacysummary.pdf. Archived at: http://www.webcitation.org/6wY3Ct9PN.

82. Federal Committee on Statistical Methodology. Statistical and Science Policy, Statistical Policy Working Paper 22, Report on Statistical Disclosure Limitation Methodology. Office of Information and Regulatory Affairs, Office of Management and Budget, 2005.[September 20, 2017], Available from: https://www.hhs.gov/sites/default/files/spwp22.pdf Archived at: http://www.webcitation.org/70cb5P3pB.
