# Supplementary material for: Automated Real-Time Collection of Pathogen-Specific Diagnostic Data: Syndromic Infectious Disease Epidemiology
Source: JMIR Public Health Surveill. 2018 Jul 6;4(3):e59. doi: 10.2196/publichealth.9876 (PMC6054708; doi:10.2196/publichealth.9876)
Supplement: Multimedia Appendix 3 [file publichealth_v4i3e59_app3.pdf]

Multimedia Appendix 3: Cleaning the Trend data

Syndromic Trends should display only valid test results acquired as part of the normal clinical testing of patients. Results from tests where an instrument, software or process control failure occurred have been removed through a combination of filters trained on data through July of 2017. In addition, there are situations in which BioFire® customers test non-patient samples: clinical customers can purchase Quality Control material from several different commercial sources and can make their own QC material by using cultured organism (from, for example, Zeptometrix, Buffalo, NY) either individually or in combination, or by using patient samples that previously tested positive for one or more organisms on the panel. Although BioFire provides recommendations for performing initial test validations [83] that involve combining pathogens in pools of 1-4 per test as well as negative controls, clinical laboratories may use other combinations of analytes. The signature of several of the commercial mixes can be identified simply by observing the set of organisms present. For example MMQC (Maine Molecular Quality Controls, Saco, ME) makes synthetic RNA mixes (M211 v1.1 and M212 v1.1) that explain 98% and 97% of the positive signals for seven and 12 analyte test results, respectively (Multimedia Appendix 3 Table, All, lines 7 and 12). The remaining 2-3% were various positive pathogens that did not match the synthetic mix profile upon comparison, potentially created by the laboratory.

Additional QC testing is associated with specific events: a site adopts a new panel, qualifies an instrument returned from repair or, more frequently, qualifies a new lot of FilmArray RP pouches. Since we cannot be sure which tests are performed on control material, we identify events that are associated with test results containing more than three positive calls and flag all tests associated with these events for removal. Multimedia Appendix 3 Figure presents an example in the case of the QC tests performed to qualify a new lot of FilmArray RP pouches. In the first six days after a new lot of FilmArray pouches is received, tests with more than three positive organism calls make up 50, 48, 18, 10, 7, and 3% of the results. Thus, for a new lot of pouches, we remove the first three days of testing to minimize the contribution from control runs, thereby deleting 1.4% of all test results from Trend.

We used the data available up to July of 2017 and the QC testing algorithms described above, to develop a set of filters that remove approximately 4% of the total tests from the full Trend

dataset which are highly enriched for QC tests. We estimate that 3% of the remaining dual and 6% of the remaining triple detection tests may still come from QC testing.

Finally we note that clinical laboratories also perform periodic proficiency testing using test samples provided by either CAP (College of American Pathologists, Northfield IL) or API (American Proficiency Institute, Traverse City, MI). This testing occurs at defined time periods during the year and BioFire is notified. Thus, we have knowledge of the organism mixes being tested as well as the number of such tests. Because only 0.05% of the results in the Trend data set are predicted to be part of CAP (Collage of American Pathologists) or API proficiency testing we have not removed them from the present study.

Because all patient identifiers are stripped before data are exported, there is no automated means to detect repeat tests on the same patient. However, the level of this testing is less than 1% of the total due to test cost and the fact that repeat testing should not be used for test-of-cure. For these reasons, the occurrence of patient duplicate testing would be rare and would not impact the overall Trend dataset. At present we cannot easily remove test results that are part of research protocols. Users at the Trend Pilot sites are instructed to add the Tag: “No Trend” to such tests (at present 0.03% of the Trend dataset) but due to the manual nature of the process we do not know how well this request is observed in practice.

References:

83. Technical Note. Guidelines for Laboratory Verification of Performance of the FilmArray® Respiratory Panel (RP). BioFire Diagnostics; 2016 [April 30, 2017] Available from: http://www.biofiredx.com/wp-content/uploads/2016/03/TN-FLM1-PRT-0060-07-Guidelines-for-Laboratory-Verification-of-Performance-of-the-FilmArray-Respiratory-Panel.pdf. Archived at: http://www.webcitation.org/6wY3OYAO6 .

Multimedia Appendix 3 Figure. QC runs performed when a new lot of FilmArray RP pouches is introduced.

Histogram of count of FilmArray RP tests with more than three positive results after a new lot of FilmArray RP pouches is introduced at a clinical site. The X-axis is the sequential number of RP tests associated with the new lot that have been performed at that site.

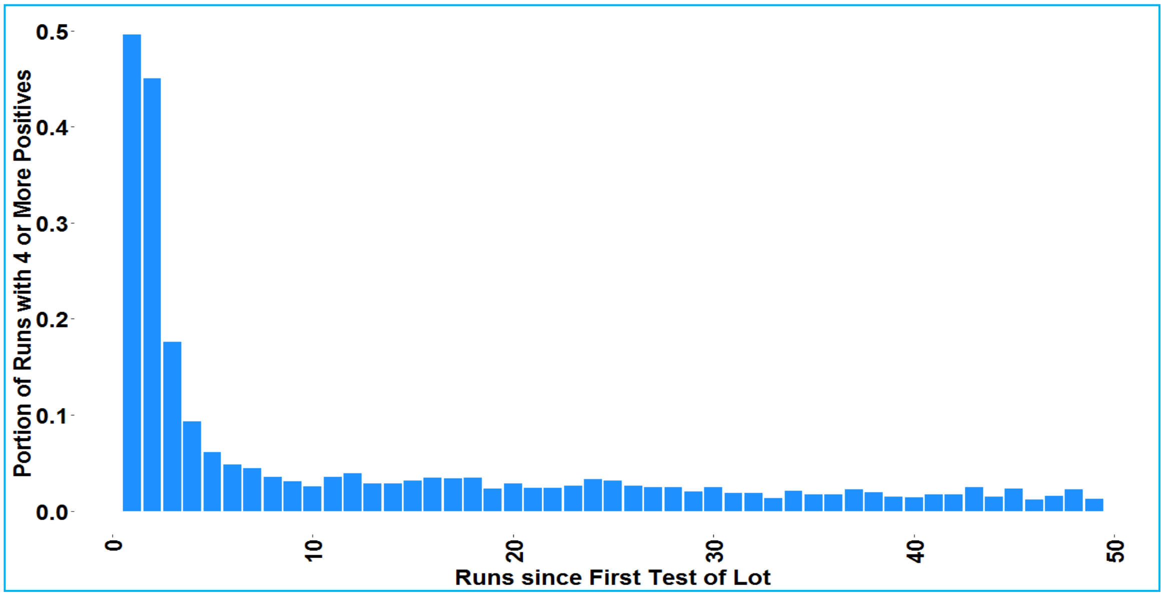

Multimedia Appendix 3 Table: Distribution of number of positive results per test for Trend RP tests

Percent of FilmArray RP tests in the Trend dataset that are positive for the indicated number of organisms for all data in the Trend dataset (All), after removing known control tests (Tests after event removal), and what is analyzed here in July 2017 (Final Trend Dataset). This analysis used Trend data up to the end of July 2017. Data are scaled to sum to 100%. The panel tests for 20 organisms but a positive for Flu A H1-2009 is reported as negative for Flu A H1 so the maximum possible number of positive results is 19.

# Positives per RP Test	All	Tests after event removal	Final Trend Dataset
0	48.16	48.47	48.91
1	42.65	43.04	43.43
2	6.64	6.65	6.71
3	0.96	0.94	0.95
4	0.19	0.16	0.00
5	0.07	0.06	0.00
6	0.06	0.04	0.00
7	0.54	0.26	0.00
8	0.01	0.01	0.00
9	0.06	0.03	0.00
10	0.03	0.02	0.00
11	0.03	0.02	0.00
12	0.50	0.26	0.00
13	0.00	0.00	0.00
14	0.01	0.00	0.00
15	0.01	0.01	0.00
16	0.03	0.02	0.00
17	0.03	0.01	0.00
18	0.02	0.00	0.00
19	0.00	0.00	0.00
