# Supplementary material for: Automated Real-Time Collection of Pathogen-Specific Diagnostic Data: Syndromic Infectious Disease Epidemiology
Source: JMIR Public Health Surveill. 2018 Jul 6;4(3):e59. doi: 10.2196/publichealth.9876 (PMC6054708; doi:10.2196/publichealth.9876)

Multimedia Appendix 4: Detection of FilmArray RP Organisms by Type

Percent detection rates aggregated from all participating Trend sites. The percent positive detection per test for each organism group is ordered by abundance.

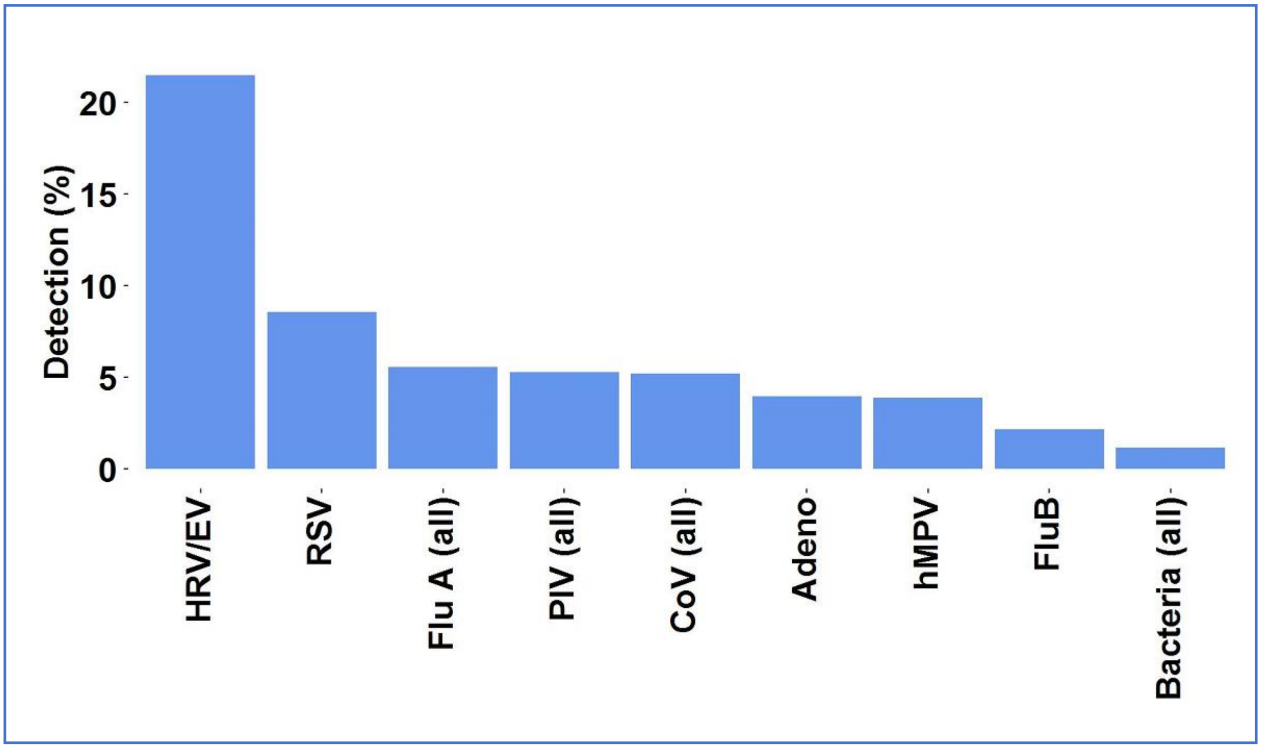

Supplement: Multimedia Appendix 4 [file publichealth_v4i3e59_app4.pdf]
