# Supplementary material for: Automated Real-Time Collection of Pathogen-Specific Diagnostic Data: Syndromic Infectious Disease Epidemiology
Source: JMIR Public Health Surveill. 2018 Jul 6;4(3):e59. doi: 10.2196/publichealth.9876 (PMC6054708; doi:10.2196/publichealth.9876)

Multimedia Appendix 5: Detection of FilmArray RP Organisms by Year

Percent detection rates from all participating Trend sites for each FilmArray RP organism are broken out by respiratory year. Organisms are ordered by total abundance across the four years.

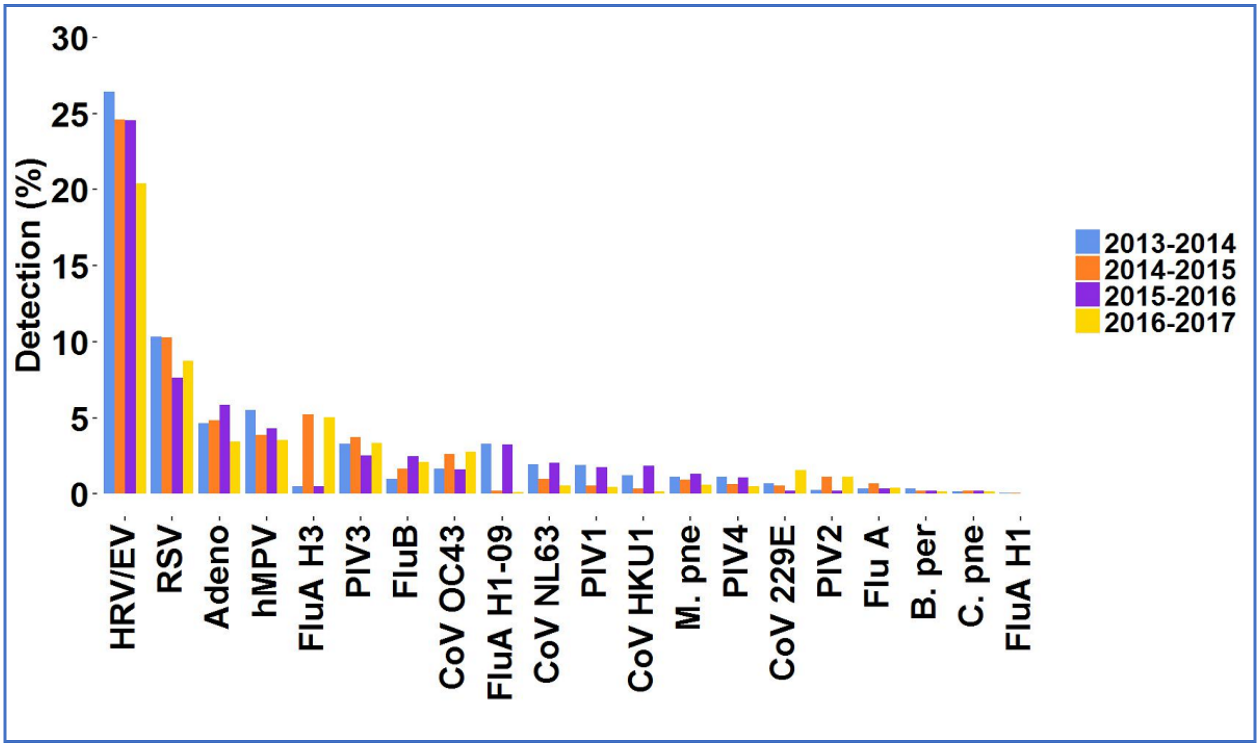

Supplement: Multimedia Appendix 5 [file publichealth_v4i3e59_app5.pdf]
