# Supplementary material for: Automated Real-Time Collection of Pathogen-Specific Diagnostic Data: Syndromic Infectious Disease Epidemiology
Source: JMIR Public Health Surveill. 2018 Jul 6;4(3):e59. doi: 10.2196/publichealth.9876 (PMC6054708; doi:10.2196/publichealth.9876)

Multimedia Appendix 6: Detection of Adenovirus and the Three Bacteria

Detection of adenovirus and the three bacterial pathogens (*B. pertussis, C. pneumoniae*, and *M. pneumoniae*) in the Trend dataset displayed as a stacked area graph. CDC measured ILI, is indicated (orange line).

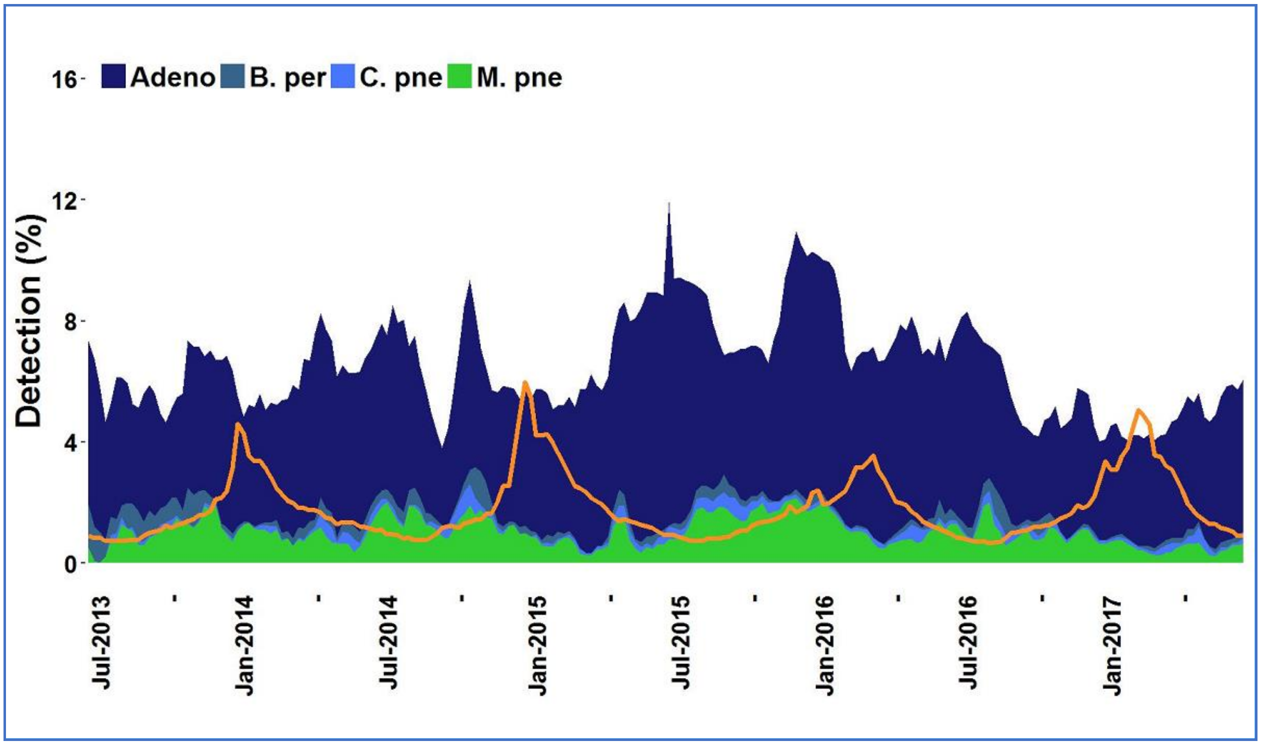

Supplement: Multimedia Appendix 6 [file publichealth_v4i3e59_app6.pdf]
