# Supplementary material for: Automated Real-Time Collection of Pathogen-Specific Diagnostic Data: Syndromic Infectious Disease Epidemiology
Source: JMIR Public Health Surveill. 2018 Jul 6;4(3):e59. doi: 10.2196/publichealth.9876 (PMC6054708; doi:10.2196/publichealth.9876)

Multimedia Appendix 7: Linear Regression of MIE and Observed Codetections

The time series data from main text figure 5, second data view, shown as a scatter plot. The equation of the linear regression is:

MIE = 4.05153 * Codetection rate - 0.0206 (R^2 value of 0.9003).

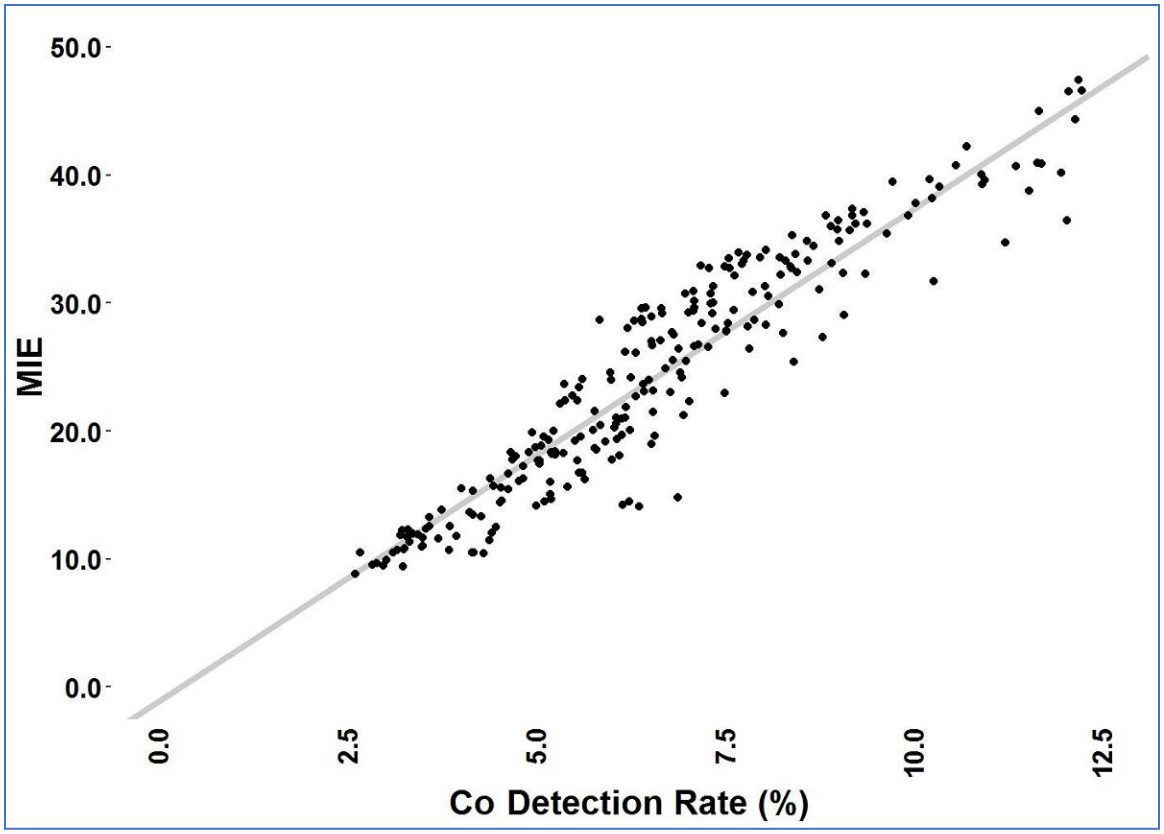

Supplement: Multimedia Appendix 7 [file publichealth_v4i3e59_app7.pdf]
